# Public Interest in Population Genetic Screening for Cancer Risk

**DOI:** 10.3389/fgene.2022.886640

**Published:** 2022-07-22

**Authors:** Megan C. Roberts, Kimberly S. Foss, Gail E. Henderson, Sabrina N. Powell, Katherine W. Saylor, Karen E. Weck, Laura V. Milko

**Affiliations:** ^1^ Division of Pharmaceutical Outcomes and Policy, UNC Eshelman School of Pharmacy, Chapel Hill, NC, United States; ^2^ Department of Genetics, University of North Carolina at Chapel Hill, Chapel Hill, NC, United States; ^3^ Department of Social Medicine, University of North Carolina at Chapel Hill, Chapel Hill, NC, United States; ^4^ Department of Medical Ethics and Health Policy, Perelman School of Medicine, University of Pennsylvania, Philadelphia, PA, United States; ^5^ Department of Pathology and Laboratory Medicine, University of North Carolina at Chapel Hill, Chapel Hill, NC, United States

**Keywords:** population genetic screening, cancer, public awareness, DNA sequencing, barriers

## Abstract

An emerging role for DNA sequencing is to identify people at risk for an inherited cancer syndrome in order to prevent or ameliorate the manifestation of symptoms. Two cancer syndromes, Hereditary Breast and Ovarian Cancer and Lynch Syndrome meet the “Tier 1” evidence threshold established by the Centers for Disease Control and Prevention (CDC) for routine testing of patients with a personal or family history of cancer. Advancements in genomic medicine have accelerated public health pilot programs for these highly medically actionable conditions. In this brief report, we provide descriptive statistics from a survey of 746 US respondents from a Qualtrics panel about the public’s awareness of genetic testing, interest in learning about their cancer risk, and likelihood of participating in a population genetic screening (PGS) test. Approximately of half the respondents were aware of genetic testing for inherited cancer risk (n = 377/745, 50.6%) and would choose to learn about their cancer risk (n-309/635, 48.7%). Characteristics of those interested in learning about their cancer risk differed by educational attainment, age, income, insurance status, having a primary care doctor, being aware of genetic testing, and likelihood of sharing information with family (*p* < 0.05). A sizeable majority of the respondents who were interested in about learning their cancer risk also said that they were likely to participate in a PGS test that involved a clinical appointment and blood draw, but no out-of-pocket cost (n = 255/309, 82.5%). Reasons for not wanting to participate included not finding test results interesting or important, concerns about costs, and feeling afraid to know the results. Overall, our results suggest that engaging and educating the general population about the benefits of learning about an inherited cancer predisposition may be an important strategy to address recruitment barriers to PGS.

## Introduction

DNA-based screening of healthy individuals has enormous, yet untapped potential to improve cancer-related health outcomes through early detection and cancer prevention before symptoms manifest. Multidisciplinary research supporting the clinical utility and validity of DNA-based population screening for certain medically actionable conditions is increasing. (Adams et al., 2016; Hunter et al., 2016; Milko et al., 2019; Zhang et al., 2019; Hendricks- Sturrup et al., 2020; Roman et al., 2020). A point of consensus for DNA-based screening is that the benefit to harm ratio can be maximized by screening for pathogenic genomic variants in well-understood causative genes for conditions with effective, evidence-based clinical interventions. (Jarvik et al., 2014; Berg et al., 2016; Green et al., 2019; Hendricks- Sturrup et al., 2020; Murray et al., 2020; Bean et al., 2021; Murray et al., 2021).

The US Centers for Disease Control and Prevention (CDC) has defined two hereditary cancer syndromes as “Tier 1” based on their clinical actionability: Lynch syndrome (LS) and Hereditary Breast and Ovarian Cancer (HBOC). Evidence indicates that population screening could significantly reduce morbidity and mortality for millions of Americans each year. (Green et al., 2019; Bean et al., 2021; Murray et al., 2021). Cost-effectiveness analyses demonstrate that screening in the general population yields good value for money and even potential cost savings for health care systems, especially when cascade screening (i.e., family testing) is considered. (Manchanda et al., 2018; Zhang et al., 2019).

The National Academies of Sciences, Engineering and Medicine’s Genomics and Public Health Action Collaborative has endorsed “an accelerated implementation science agenda” (Khoury et al., 2018) for the Tier 1 conditions, including LS, HBOC, and familial hypercholesterolemia (FH), to understand the potential impact of population genomic screening in healthy adults. Increasingly, these three conditions and eleven associated genes are being adopted for Population Genetic Screening (PGS) pilot programs to investigate clinical and implementation outcomes in various health care settings around the country. (Brown-Johnson et al., 2021; Christensen et al., 2021; East et al., 2021).

Though the capacity for clinical PGS programs to transform personalized public health in the United States is widely acknowledged (Evans et al., 2013; Green et al., 2020), current public interest in participating in PGS is an essential yet understudied aspect of equitable implementation. Several studies examining the public’s interest in population genetic screening have shown that awareness about genetic screening for certain types of cancer is associated with being non-Hispanic White (Hay et al., 2018; Rubinsak et al., 2019), willingness to pay for testing, having a family history of cancer, and higher educational attainment. (Shen et al., 2022). Other population characteristics such as access to a primary care provider, rurality, income, insurance, sexual orientation, and gender have been shown to be related to genetic services use; however little is understood about their association with interested in and likelihood to participate in PGS. To this end, we conducted a survey to more comprehensively understand the characteristics of those who are interested in participating in PGS for learning about cancer risk. The results of this study may inform strategies to increase awareness and participation in PGS programs among diverse populations.

## Methods

### Population

In December 2020, the UNC Lineberger Cancer Prevention and Control Program recruited an online convenience sample of US adults through the Qualtrics Online Panel platform (n = 746, Qualtrics, Seattle WA). Participants were eligible if they were over the age of 18 and resided in the US. Qualtrics panel members are recruited from multiple sources including but not limited to, targeted email lists, gaming sites, customer loyalty web portals, and social media. Panel members were sent an email invitation or were prompted on the survey platform to respond to the online survey for a specific compensation amount. Because panel members can be reimbursed in different ways (e.g., gather points, donate funds, etc.), incentive amount for this study is estimated to be $2.50 per person. Interested respondents clicked on the survey link provided. The University of North Carolina Institutional Review Board approved this study (#20–2338).

### Measures

Primary outcomes of interest were adapted from existing surveys and included:

(1) awareness about genetic tests (i.e., *Genetic tests that analyze your DNA for potential cancer risks are currently available. Have you heard or read about these genetic tests? Yes/no*) (HINTS, 2022);
(2) receipt of genetic testing (among those aware of genetic testing: *Have you ever had a genetic test to determine if you have an increased risk of developing cancer? Yes/no*) (HINTS, 2022);
(3) interest in learning genetic risk (among those who have not received genetic testing: *How interested would you be in learning whether you have a genetic risk factor for cancer that can be prevented or treated? Likert 1–5*) (Peterson et al., 2022);
(4) likelihood of getting a PGS test (among those who were interested in learning genetic risk: *To learn whether you have a genetic risk factor for cancer, you would need to make an appointment at a local clinic, get your blood drawn, and set up an online account to access your test results. Assuming there is no cost to you, how likely would you be to get this test?* Likert 1–5); and
(4a) reasons why you selected “unlikely/very unlikely” or “neither likely or unlikely” or “likely/very likely” (open-ended response). (See Supplementary Figure S1).

Given that cost is a known barrier to genetic testing among patients (Steffen et al., 2017), we asked specifically about interest in a screening test that would be at no-cost to patients. Participants were also asked to explain how they arrived at their answer about their likelihood of getting a PGS test in an open field question (question 4a above).

Because we were interested in understanding which subpopulations are aware of, engaged in or interested in engaging in PGS programs, we collected sociodemographic information that has been associated with awareness about or use of genetic services in prior studies. These characteristics included: gender (women/men) (Sanderson et al., 2004; Childers et al., 2018), ethnicity (Hispanic, non-Hispanic) (Childers et al., 2018; Salloum et al., 2018), race (white, black, other) (Salloum et al., 2018; Chapman-Davis et al., 2021), education (less than HS/HS/GED, some college/technical school, AD, BS, graduate/professional degree) (Sanderson et al., 2004; Armstrong et al., 2005; Childers et al., 2018), age (<25, 25–49, 50–74, 75+) (Sanderson et al., 2004; Orlando et al., 2019), income ($0–34,999, $35,000–99,999, $100K+) (Armstrong et al., 2005), insurance (Medicare/Medical Assistance/any kind of government-assistance plan for those with low incomes or a disability, Employer-based, any Medicaid, and other) (The National Academies Collection: Reports funded by National Institutes of Health, 2018), sexual orientation (straight, gay or lesbian, bisexual, prefer to self-describe) (Nathan et al., 2019), and rurality (urban, suburban, rural). (Salloum et al., 2018). We also included several additional variables: having a primary care doctor (yes/no), (Armstrong et al., 2005), perceived comparative cancer risk (5 point Likert scale) (Chopra and Kelly, 2017), and intentions to share results with family (8 point Likert scale) (Chopra and Kelly, 2017). These variables were included as conversations with primary care providers, (Armstrong et al., 2005), having higher perceived cancer risk (often due to family or personal cancer history) (Chopra and Kelly, 2017), and intentions to share results with family members have been associated with increased genetic services use (Chopra and Kelly, 2017).

### Analysis

We calculated descriptive statistics to examine whether there were differences between the characteristics of respondents according to our primary outcomes. We used chi-square tests to examine differences between those who were 1) aware of genetic tests versus not aware, 2) previously tested versus not tested, 3) interested (very interested, interested) in PGS versus not interested (neutral, uninterested, very uninterested), and 4) likely to participate in PGS versus not likely (neutral, unlikely, very unlikely).

We used thematic analysis to understand respondents free text responses to their likelihood of participating in PGS (question 4a above). We report the top five themes among those who were likely, unlikely, and neutral about getting a PGS test (MCR and LVM). Independently coders reviewed open-ended responses and identified emergent themes for each response category (unlikely: unlikely/very unlikely; neutral: neither likely or unlikely; likely: likely/very likely) and applied them to 10% of responses in each category. Coders compared themes and their application, modified the list of themes, and then independently applied the codes to 20% of responses (n = 60). We repeated this process until we achieved 100% agreement (in one round), and then we independently coded the remaining responses. We calculated the level of agreement between coders (87.6%). Conflicts were then reconciled through discussion. We reported the major themes and exemplar quotes.

## Results

Of the overall sample of respondents, 377 (50.6%) were aware of genetic tests. Among those who were aware of genetic tests, a higher proportion were non-Hispanic, in the middle age categories, as well as had higher educational attainment, Medicare or other insurance, a primary care doctor and higher perceived comparative cancer risk. No significant differences between gender, race, income, rurality, and sexual orientation were identified (Table 1).

**TABLE 1:** Overall descriptive statistics and by awareness about genetic tests.

Characteristics	Survey item: *Genetic tests that analyze your DNA for potential cancer risks are currently available. Have you heard or read about these genetic tests?*						
	Total		No		Yes		*p*
	N	%	N	%	n	%	
Overall	745^a^	—	368	49.4	377	50.6	—
Gender^b^	—	—	—	—	—	—	0.43
Women	436	58.52	216	58.70	220	58.36	—
** **Men	287	38.52	139	37.77	148	39.26	—
Ethnicity	—	—	—	—	—	—	**0.01**
** **non-Hispanic	644	86.44	306	83.15	338	89.66	—
** **Hispanic	101	13.56	62	16.85	39	10.34	—
Race	—	—	—	—	—	—	0.11
** **White	569	76.38	269	73.10	300	79.58	—
** **Black	78	10.47	43	11.68	35	9.28	—
Other	98	13.15	56	15.22	42	11.14	—
Education	—	—	—	—	—	—	**<0.01**
** **Less than H*S or HS/GED*	187	25.10	114	30.98	73	19.36	—
** **Some college/technical school	153	20.54	79	21.47	74	19.63	—
** **AD	85	11.41	31	8.42	54	14.32	—
** **BS	175	23.49	84	22.83	91	24.14	—
** **Graduate/professional degree	145	19.46	60	16.30	85	22.55	—
Age	—	—	—	—	—	—	**0.001**
** **<25	126	17.10	76	20.94	50	13.37	—
** **25–49	333	45.18	166	45.73	167	44.65	—
** **50–74	247	33.51	100	27.55	147	39.30	—
** **≥75	31	4.21	21	5.79	10	2.67	—
Income	—	—	—	—	—	—	0.08
** **0–34,999	310	41.67	168	45.78	142	37.67	—
** **35,000–99,999	267	36.16	123	33.51	146	38.73	—
** **100,000+	165	22.18	76	20.71	89	23.61	—
Insurance	—	—	—	—	—	—	**<0.001**
** **Any Medicaid/Aid	253	34.10	124	33.97	129	34.22	—
** **Medicare	185	24.93	73	20.00	112	29.71	—
** **Employer-based	130	17.52	74	20.27	56	14.85	—
Other^c^	81	10.92	34	9.32	47	12.47	—
No Insurance	93	12.53	60	16.44	33	8.75	—
Rurality	—	—	—	—	—	—	0.14
** **Urban	251	33.74	135	36.78	116	30.77	—
** **Suburban	342	45.97	166	45.23	176	46.68	—
** **Rural	151	20.30	66	17.98	85	22.55	—
Have a primary doctor?	—	—	—	—	—	—	**<0.001**
** **Yes	416	55.84	174	47.28	242	64.19	—
** **No	329	44.16	194	52.72	135	35.81	—
Sexual Orientation^b^	—	—	—	—	—	—	0.88
** **Straight	650	87.37	322	87.50	328	87.23	—
** **Gay or lesbian	34	4.57	17	4.62	17	4.52	—
** **Bisexual	47	6.32	24	6.52	23	6.12	—
Comparative Cancer Risk^d^	—	—	—	—	—	—	**<0.01**
** **Very unlikely	80	12.64	51	15.94	29	9.27	—
** **Unlikely	118	18.64	63	19.69	55	17.57	—
** **Neither likely or unlikely	276	43.60	138	43.13	138	44.09	—
** **Likely	109	17.22	53	16.56	56	17.89	—
** **Very likely	50	7.90	15	4.69	35	11.18	—

a One survey respondent did not answer this item (n = 745); Because of missing data, not all column numbers add to 745 (n = 14, 0.2% missing data fields).

b Other categories were censored due to small cell size; column percentages will not sum to 100%.

c Other insurance (Tricare, VA, HIS, self-pay, other).

d Those who reported a personal history of cancer did not receive this item (total responses = 633).

Bold indicates a *p*-value of < 0.05.

Among those aware of genetic tests, 110 respondents (29.2%) had received a genetic test for an inherited cancer risk in the past. Compared to the 267 respondents who had not previously received a genetic test, the tested group had a higher proportion of men, higher educational attainment, higher income, had insurance coverage, lived in urban areas, were in the middle age categories, and identified as not straight (Table 2).

**TABLE 2:** Descriptive characteristics of those how have and have not received a genetic test for cancer risk.

Characteristics	Survey item: ^a^ *Have you ever had a genetic test to determine if you have an increased risk of developing cancer?*				
	No		Yes		*p*
	n	%	N	%	
Overall	267	70.82	110	29.18	—
Gender^b^	—	—	—	—	**<0.01**
** **Women	171	64.04	49	44.55	—
** **Men	92	34.46	56	50.91	—
Ethnicity	—	—	—	—	0.55
** **non-Hispanic	241	90.26	97	88.18	—
** **Hispanic	26	9.74	13	11.82	—
Race	—	—	—	—	0.19
** **White	219	82.02	81	73.64	—
** **Black	22	8.24	13	11.82	—
Other	26	9.74	16	14.55	—
Education	—	—	—	—	**<0.001**
** **Less than HS or HS/GED	53	19.85	20	18.18	—
** **Some college/technical school	65	24.34	—	—	—
** **AD	43	16.10	11	10.00	—
** **BS	60	22.47	31	28.18	—
** **Graduate/professional degree	46	17.23	39	35.45	—
Age	—	—	—	—	**<0.001**
** **<25	32	12.08	18	16.51	—
** **25–49	96	36.23	71	65.14	—
** **50–74	127	47.92	20	18.35	—
** **≥75	10	3.77	—	—	—
Income	—	—	—	—	**<0.001**
** **0–34,999	109	40.82	33	30.00	—
** **35,000–99,999	115	43.07	31	28.18	—
** **100,000+	43	16.10	46	41.82	—
Insurance	—	—	—	—	**<0.01**
** **Any Medicaid	75	28.09	54	49.09	—
** **Medicare	83	31.09	29	26.36	—
** **Employer-based	45	16.85	11	10.00	—
Other^c^	35	13.11	12	10.91	—
No Insurance	29	10.86	—	—	—
Rurality	—	—	—	—	**<0.001**
** **Urban	64	23.97	52	47.27	—
** **Suburban	134	50.19	42	38.18	—
** **Rural	69	25.84	16	14.55	—
Have a primary doctor?	—	—	—	—	0.13
** **Yes	165	61.80	77	70.00	—
** **No	102	38.20	33	30.00	—
Sexual Orientation^b^	—	—	—	—	**<0.01**
** **Straight	241	90.60	87	79.09	—
** **Gay or lesbian	—	—	10	9.09	—
** **Bisexual	11	4.14	12	10.91	—
Comparative Cancer Risk^d^	—	—	—	—	0.21
** **Very unlikely	23	9.62	--	--	
** **Unlikely	40	16.74	15	20.27	
** **Neither likely or unlikely	113	47.28	25	33.78	
** **Likely	40	16.74	16	21.62	
** **Very likely	23	9.62	12	16.22	

a Among survey respondents who were aware of genetic tests (n = 377); Because of missing data, not all column numbers add to 377 (n = 8, 0.2% missing data fields).

b Other categories were censored due to small cell size; column percentages will not sum to 100%.

c Other insurance (Tricare, VA, HIS, self-pay, other).

d Those who reported a personal history of cancer did not receive this item (total responses = 313).

Cell sizes less than 10 are not reported. Bold indicates a *p*-value of < 0.05.

Of the 635 respondents who had not received genetic test, 309 (48.7%) would be interested in learning whether they had a genetic risk factor for cancer. Among those interested in learning about their risk, a higher proportion were aware of genetic tests, had higher income, had a primary care doctor, had insurance coverage, were in older age categories, had higher educational attainment, and would be more likely to share information with family (Table 3).

**TABLE 3:** Descriptive characteristics among those with different levels of interest in learning about genetic cancer risks.

Characteristics	Survey item: *How interested would you be in learning whether you have a genetic risk factor for cancer that can be prevented or treated? (n = 635)* ^a^						
	^b^not interested		Neutral		Interested		*P*
	n	%	n	%	n	%	
Overall	183	28.82	143	22.52	309	48.66	—
Gender^c^	—	—	—	—	—	—	0.16
** **Women	103	56.28	94	65.73	190	61.49	—
** **Men	74	40.44	43	30.07	114	36.89	—
Ethnicity	—	—	—	—	—	—	0.13
** **non-Hispanic	152	83.06	120	83.92	275	89.00	—
** **Hispanic	31	16.94	23	16.08	34	11.00	—
Race	—	—	—	—	—	—	0.21
** **White	135	73.77	105	73.43	248	80.26	—
** **Black	18	9.84	16	11.19	31	10.03	—
Other	30	16.39	22	15.38	30	9.71	—
Education	—	—	—	—	—	—	**0.04**
** **Less than HS or HS/GED	51	27.87	49	34.27	67	21.68	—
** **Some college/technical school	42	22.95	36	25.17	66	21.36	—
** **AD	19	10.38	15	10.49	40	12.94	—
** **BS	38	20.77	31	21.68	75	24.27	—
** **Graduate/professional degree	33	18.03	12	8.39	61	19.74	—
Age	—	—	—	—	—	—	**0.04**
** **<25	35	19.44	32	22.70	41	13.36	—
** **25–49	61	33.89	55	39.01	146	47.56	—
** **50–74	73	40.56	49	34.75	105	34.20	—
** **≥75	11	6.11	—	—	15	4.89	—
Income	—	—	—	—	—	—	**<0.001**
** **0–34,999	90	49.18	79	55.63	108	34.95	—
** **35,000–99,999	70	38.25	47	33.10	121	39.16	—
** **100,000+	23	12.57	16	11.27	80	25.89	—
Insurance	—	—	—	—	—	—	**<0.001**
** **Any Medicaid	49	26.78	40	28.37	110	35.71	—
** **Medicare	54	29.51	26	18.44	76	24.68	—
** **Employer-based	24	13.11	25	17.73	70	22.73	—
Other^d^	24	13.11	15	10.64	30	9.74	—
No Insurance	32	17.49	35	24.82	22	7.14	—
Rurality	—	—	—	—	—	—	0.19
** **Urban	53	28.96	36	25.35	110	35.60	
** **Suburban	90	49.18	76	53.52	134	43.37	
** **Rural	40	21.86	30	21.13	65	21.04	
Have a primary doctor?							**<0.001**
** **Yes	81	44.26	60	41.96	198	64.08	
** **No	102	55.74	83	58.04	111	35.92	
How likely to share with family	—	—	—	—	—	—	**<0.001**
** **Not at all likely	56	30.60	—	—	11	3.56	—
** **Not likely	16	8.74	—	—	—	—	—
** **Somewhat not likely	24	13.11	16	11.19	—	—	—
** **Neither likely or unlikely	27	14.75	78	54.55	25	8.09	—
** **Somewhat likely	23	12.57	23	16.08	59	19.09	—
** **Likely	14	7.65	11	7.69	66	21.36	—
** **Very likely	23	12.57	10	6.99	134	43.37	—
Aware of genetic tests	70	38.25	51	35.7	146	47.2	**0.03**

a Among those who reported not receiving a genetic test; Because of missing data, not all column numbers add to 635 (n = 12, 0.2% missing data fields).

^b^Not interested = not at all interested, not interested, somewhat not interested; Neutral = neither interested or uninterested; Interested = very interested, interested, somewhat interested.

^c^We did not report “prefer not to say” for gender given small cell size, nor do we present sexual orientation.

^d^Other insurance (Tricare, VA, HIS, self-pay other).

Cell sizes less than 10 are not reported.

Bold indicates a *p*-value of < 0.05.

Finally, of 309 respondents interested in learning whether they had a genetic risk factor for cancer, a substantial majority, 255 respondents (82.5%), would be likely or highly likely to get a PGS test (Table 4). Within this group, a higher proportion were male, in middle age categories, and were more likely to share information with family. Major themes emerged for being likely to get a PGS test and they included 1) believing the test could inform their health and/or plan for the future (n = 51 of 255), 2) wanting to know their risk of cancer (n = 46 of 255), 3) finding the test “important” (n = 45 of 255), 4) finding the test easy/available/free (n = 35 of 255), 5) having a family history of cancer (n = 21 of 255), and 6) having a personal history of cancer or other risk factors for cancer (n = 11 of 255). Only 14 respondents (4.5%) were unlikely to get a PGS test, and the top reasons were 1) not being interested or finding the test important (n = 5), 2) concerns about costs (n = 2), and 3) not wanting to or being scared to know (n = 2). The 40 respondents who were neutral (12.9%) were not sure if they wanted the test yet (n = 8), concerned about logistics (n = 4), felt the test was not interesting or important for them (n = 4), among other less common reasons (See Supplementary Table S1).

**TABLE 4:** Descriptive characteristics among those with different likelihoods of getting a population genetic screening test for cancer risk.

Characteristics	Survey item: ^a^ *To learn whether you have a genetic risk factor for cancer, you would need to make an appointment at a local clinic, get your blood drawn, and set up an online account to access your test results. Assuming there is no cost to you, how likely would you be to get this test? (n = 309)*						
	^b^unlikely to get Test		Neutral		Likely to get Test		*p*
	N	%	n	%	n	%	
Overall	14	4.53	40	12.94	255	82.52	—
Gender^c^	—	—	—	—	—	—	**<0.001**
** **Women	—	—	28	70.00	157	61.57	—
** **Men	—	—	12	30.00	97	38.04	—
Ethnicity	—	—	—	—	—	—	0.86
** **non-Hispanic	12	85.71	35	87.50	228	89.41	—
** **Hispanic	—	—	—	—	27	10.59	—
Race	—	—	—	—	—	—	0.09
** **White	10	71.43	30	75.00	208	81.57	
** **Black	—	—	—	—	26	10.20	—
Other	—	—	—	—	21	8.24	—
Education	—	—	—	—	—	—	0.09
** **Less than HS or HS/GED	—	—	14	35.00	48	18.82	—
** **Some college/technical school	—	—	10	25.00	51	20.00	—
** **AD	—	—	—	—	34	13.33	—
** **BS	—	—	—	—	66	25.88	—
** **Graduate/professional degree	—	—	—	—	56	21.96	—
Age	—	—	—	—	—	—	**0.001**
** **<25	—	—	12	30.00	25	9.88	—
** **25–49	—	—	14	35.00	130	51.38	—
** **50–74	—	—	10	25.00	89	35.18	—
** **≥75	—	—	—	—	—	—	—
Income	—	—	—	—	—	—	0.10
** **0–34,999	—	—	21	52.50	81	31.76	—
** **35,000–99,999	—	—	10	25.00	105	41.18	—
** **100,000+	—	—	—	—	69	27.06	—
Insurance	—	—	—	—	—	—	0.07
** **Any Medicaid	—	—	14	35.00	90	35.43	—
** **Medicare	—	—	10	25.00	61	24.02	—
** **Employer-based	—	—	—	—	66	25.98	—
Other^d^	—	—	—	—	22	8.66	—
No Insurance	—	—	—	—	15	5.91	—
Rurality	—	—	—	—	—	—	0.14
** **Urban	—	—	13	32.50	93	36.47	—
** **Suburban	—	—	22	55.00	108	42.35	—
** **Rural	—	—	—	—	54	21.18	—
Have a primary doctor?	—	—	—	—	—	—	0.23
** **Yes	—	—	27	67.50	165	64.71	—
** **No	—	—	13	32.50	90	35.29	—
How likely to share with family	—	—	—	—	—	—	**<0.01**
** **Not at all likely	—	—	—	—	—	—	—
** **Not likely	—	—	—	—	—	—	—
** **Somewhat not likely	—	—	—	—	—	—	—
** **Neither likely or unlikely	—	—	—	—	18	7.06	—
** **Somewhat likely	—	—	12	30.00	46	18.04	—
** **Likely	—	—	—	—	62	24.31	—
** **Very likely	—	—	13	32.50	113	44.31	—
Aware of genetic tests	—	—	16	40.00	124	48.63	0.56

a Marked somewhat-very interested in “learning whether you have a genetic risk factor for cancer that can be prevented or treated (n = 309 of 635 who have not yet received genetic testing); Because of missing data, not all column numbers add to 309 (n = 3, 0.1% missing data fields).

b Unlikely to get test = very unlikely, unlikely; Neutral = neither likely nor unlikely; Likely to get test = very likely, likely.

c Other categories were censored due to small cell size; column percentages will not sum to 100%.

d Other insurance (Tricare, VA, HIS, self-pay, other).

Cell sizes less than 10 are not reported.

Bold indicates a *p*-value of < 0.05.

## Discussion

Despite increasing availability of direct-to-consumer (DTC) testing and PGS programs, we found that awareness of genetic testing for cancer predisposition in the general population remains at around 50%. This aligns with prior research from 2017 from the National Cancer Institute (NCI) Health Information National Trends Survey in which 57% of respondents reported being aware of genetic tests used for health reasons. (Roberts et al., 2019). This percentage is higher than a decade ago, at which time awareness about DTC genetic testing was 38.1% (Apathy et al., 2018). Our results were also consistent with prior reports of the association between awareness of genetic testing and education level, (Sanderson et al., 2004; Armstrong et al., 2005; Childers et al., 2018), demonstrating a persistent need to reach individuals with lower educational attainment to prevent widening disparities in access to precision health care.

Though racial disparities in genetic testing utilization are well established in the literature, (Salloum et al., 2018; Chapman-Davis et al., 2021), we did not find statistically significant disparities in our data, likely because only individuals who reported being aware of genetic tests were asked about their genetic testing history. This aligns with data from the NCI using a similar measure about awareness of DTC genetic testing in which differences by race were not observed. (Agurs -Collins et al., 2015). We did find that people of Hispanic ethnicity were significantly less likely to be aware of testing, indicating that efforts to increase accessibility to precision health care should also include native Spanish speakers.

Among those aware of genetic testing who have not already had genetic testing, almost half would be interested in learning whether they had a genetic risk factor for cancer that can be prevented or treated, which is lower than what has been reported elsewhere in public samples. (Donovan and Tucker, 2000; Alvord et al., 2020). For example, in a 2020 study of public perception of predictive cancer genetic testing in Oregon, 87% of participants reported an interest in cancer genetic testing and receiving genetic information about themselves; however, it is important to note that 85% of individuals in this study had a personal or family diagnosis of cancer. As better understanding of family cancer risks is a known motivator for testing reported by participants in that study, this study also highlights an urgent need for more data from participants with no prior personal or family history of cancer. (HINTS, 2019). In our data we found that respondents who were likely to share their results with family were also more likely to be interested in learning whether they had a genetic risk factor for cancer. Deeper understanding about the reasons for overall disinterest in testing (e.g. uninformed about benefits vs mistrust of health system) will be important for developing strategies to engage the broader population in genetic screening. We also found socioeconomic factors (educational attainment, insurance, income), age, and having a primary care provider differed, such that larger proportions of those who are traditionally underserved and those without a primary care provider reported being uninterested in learning about their genetic risk for cancer. This aligns with prior work that has demonstrated potential disparities in genetic services use among these populations. (Shen et al., 2022; Sanderson et al., 2004; Childers et al., 2018; Armstrong et al., 2005; Orlando et al., 2019; The National Academies Collection: Reports funded by National Institutes of Health, 2018).

Among respondents who had not had any previous genetic testing, about half were interested in learning about a genetic risk for cancer predisposition. Furthermore, a large majority said they would be likely to participate in a PGS test in a clinical setting that required making an appointment, getting a blood draw, and creating an online account for a patient portal. All respondents provided contextual information about how and why they responded to this question and, interestingly, only one respondent mentioned mistrust or concerns about genetic discrimination, data privacy or security, which have been commonly reported in the literature. (Hann et al., 2017). This may be due to our small sample size of individuals who reported being very unlikely or unlikely to participate in the PGS (n = 14) and also that these concerns are reflected in the high proportion of respondents who were not interested in learning about a genetic risk for cancer predisposition. Overall, we found it telling that most survey respondents who were interested in learning their risk for developing an inherited cancer syndrome would also hypothetically be willing to commit the time and effort participate in a clinical offering that included a blood draw and a patient portal. Of note, cost was still a concern of respondents despite explicitly noting that the hypothetical clinical screening test would be at no-cost to patients. A deeper awareness about participants’ downstream financial concerns is warranted and may help us better understand barriers related to follow-up medical costs or costs associated with taking time off to get a blood draw.

While these descriptive findings provide foundational data on public awareness, use and interest in genetic testing and screening, they should be interpreted within the context of several limitations. First, because of small sample size, we are unable to examine multivariable associations between key variables and certain outcomes (receipt of a genetic test, interest in learning genetic risk and likelihood of getting a PGS test). Further, while Qualtrics survey samples can provide a diverse sample, (Miller et al., 2020), respondents may not be representative of the general population. We used convenience sampling from a geographically diverse area in the US to rapidly gather data about the public’s opinions and to generate hypotheses about potentially important factors related to stakeholder engagement around PGS. However, this sampling approach has several drawbacks including lack of generalizability, as well as selection, sampling, and positivity biases. Further, we are unable to determine the denominator required to calculate a response rate given the ways in which participants were invited to take the survey. Future work should include larger nationally representative samples to better understand the association between key sociodemographic variables and key outcomes which will be essential for ensuring equity in the implementation of population genetic screening program. In addition, we asked about individuals’ *intentions* to learn about genetic risk and *likelihood of participating* in a PGS test. Intentions are associated with health behaviors, as are other factors, some of which are known, such as perceived control (data not collected) and others which are less certain. (Ajzen, 1991). Thus, it will be important for clinical PGS programs to collect data on actual uptake of PGS to understand how the public engages with PGS in real world settings. This work should be expanded to include other clinical contexts, such as familial hypercholesterolemia, which is also a CDC Tier One condition for which population genetic screening has the potential to improve precision public health. (CDC, 2021). Because we test multiple comparisons, our chances of having a type I error are higher; results should be interpreted in this light. Finally, the population genetic screening program described in the survey item mentioned that the test would be free, clinic-based, and require a blood draw, limiting the generalizability of our findings to PGS programs with different characteristics. Future studies to compare different PGS models will further inform the implementation of PGS models moving forward.

Our findings identified two key challenges for the implementation of population genetic screening: increasing awareness of the potential benefits of genetic testing and interest in learning one’s genetic risk for cancer. This may be especially important for subpopulations with lower socioeconomic status and those without a primary source of care. Future work to better understand and develop strategies to overcome these challenges will be essential as PGS programs are increasingly implemented into clinical practice.

## Data Availability

The raw data supporting the conclusions of this article will be made available by the authors, without undue reservation.

## Supplementary Material

Supplementary Figure S1 Survey flow and primary measures of interest.

Supplementary Table S1 Open-ended responses about reasons for respondents’ perceived likelihood of getting a population genetic screening test.
