# Supplementary figures and images for: Public Interest in Population Genetic Screening for Cancer Risk
Source: Front Genet. 2022 Jul 22;13:886640. doi: 10.3389/fgene.2022.886640 (PMC9354961; doi:10.3389/fgene.2022.886640)

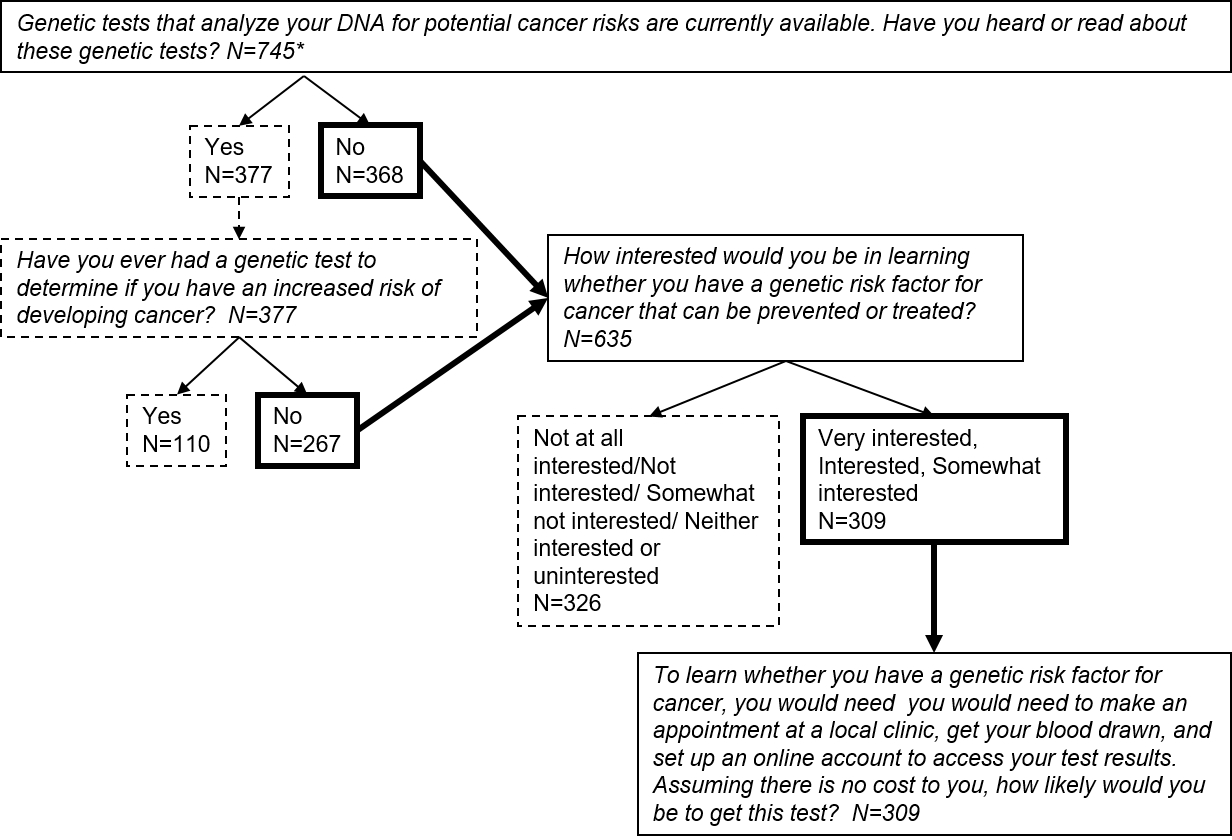

Supplement: Supplementary file 2 [file Image1.png]
